# Comparative transcriptome analysis of heat stress responses of *Clematis lanuginosa* and *Clematis crassifolia*

**DOI:** 10.1186/s12870-022-03497-w

**Published:** 2022-03-23

**Authors:** Renjuan Qian, Qingdi Hu, Xiaohua Ma, Xule Zhang, Youju Ye, Hongjian Liu, Handong Gao, Jian Zheng

**Affiliations:** 1 College of Forestry, Nanjing Forestry University, Nanjing, 210037 China; 2 Wenzhou Key Laboratory of Resource Plant Innovation and Utilization, Zhejiang Institute of Subtropical Crops, Wenzhou, 325005 Zhejiang China; 3 Zhejiang Institute of Subtropical Crops, Zhejiang Academy of Agricultural Sciences, Zhejiang 310021 Wenzhou, China

**Keywords:** Heat stress, Transcriptome, Pathway analysis, *Clematis lanuginosa*, *Clematis crassifolia*

## Abstract

**Background:**

*Clematis* species are attractive ornamental plants with a variety of flower colors and patterns. Heat stress is one of the main factors restricting the growth, development, and ornamental value of *Clematis*. *Clematis lanuginosa* and *Clematis crassifolia* are large-flowered and evergreen *Clematis* species, respectively, that show different tolerance to heat stress. We compared and analyzed the transcriptome of *C. lanuginose* and *C. crassifolia* under heat stress to determine the regulatory mechanism(s) of resistance.

**Results:**

A total of 1720 and 6178 differentially expressed genes were identified from *C. lanuginose* and *C. crassifolia*, respectively. The photosynthesis and oxidation–reduction processes of *C. crassifolia* were more sensitive than *C. lanuginose* under heat stress. Glycine/serine/threonine metabolism, glyoxylic metabolism, and thiamine metabolism were important pathways in response to heat stress in *C. lanuginose*, and flavonoid biosynthesis, phenylalanine metabolism, and arginine/proline metabolism were the key pathways in *C. crassifolia*. Six *sHSP*s (*c176964_g1, c200771_g1, c204924_g1, c199407_g2, c201522_g2, c192936_g1*), *POD1* (*c200317_g1*), *POD3* (*c210145_g2*), *DREB2* (*c182557_g1*), and *HSFA2* (*c206233_g2*) may be key genes in the response to heat stress in *C. lanuginose* and *C. crassifolia*.

**Conclusions:**

We compared important metabolic pathways and differentially expressed genes in response to heat stress between *C. lanuginose* and *C. crassifolia*. The results increase our understanding of the response mechanism and candidate genes of *Clematis* under heat stress. These data may contribute to the development of new *Clematis* varieties with greater heat tolerance.

**Supplementary Information:**

The online version contains supplementary material available at 10.1186/s12870-022-03497-w.

## Background

*Clematis* L. (Ranunculaceae) are mainly perennial woody vines. There are many species, varieties, and flower patterns. The optimum growth conditions for *Clematis* generally involve cool to moderate temperatures. There are approximately 355 species of *Clematis* worldwide and more than 147 species in China [1, 2]. The horticultural *Clematis* varieties are mostly cultivated in Poland, Britain, and other European countries.

*Clematis* species also have medicinal value. Their chemical components include triterpenoid saponins, alkaloids, flavonoids, lignans, and steroids. *Clematis* spp. are plant sources of many pharmaceutical active ingredients [3–5]. Flavonoids isolated from *Clematis aethusifolia* showed moderate toxicity to five human solid tumor cell lines, including A-375 and SK-OV-3 [6]. The triterpene saponins isolated from *Clematis aethusifolia* inhibited the growth and development of *Plutella xylostella* in crops, and may have potential for insecticide development [7]. *Clematis* is also an important genus of garden greening vines that increases the diversity of vertical greening plants in gardens. They can be used as ground cover plants, potted plants, and garden greening plants, and are excellent ornamentals.

With global climate change and increased temperatures, extreme heat stress has become a problem that threatens plant growth and development. Heat stress can cause physiological, molecular, and biochemical changes in plants and interfere with the growth and development process of cells and the entire plant. Heat stress can limit the growth, metabolism, and yield of plants [8–10]. Heat stress affects seed germination and growth, leading to seed malformation and seed cell death. Heat stress also affects the development of flower organs [11, 12]. Some horticultural varieties of *Clematis* show symptoms such as leaf wilting and plant wilt after suffering from heat stress. Heat stress significantly restricts the ornamental uses and growth environment of *Clematis*. Therefore, understanding the heat resistance mechanism of *Clematis* and the breeding of heat-resistant varieties are important topics.

Plants can adapt to, or resist, heat stress by morphological changes, photosynthesis, protective enzyme activity, and osmotic substance regulation [13, 14]. Plant antioxidant activity is mainly completed by the enzymatic and non-enzymatic clearance systems. Enzymatic clearance systems include superoxide dismutase (SOD), catalase (CAT), and peroxidase (POD); non-enzymatic clearance systems include ascorbic acid (ASA), mannitol (MT), and vitamin E. When the plant suffers from heat stress, the balance of reactive oxygen radicals in the plant will be disrupted, reactive oxygen species (ROS) accumulation will occur, and the reactive oxygen scavenging system will help to reduce the damage caused by the stress [15–17]. The plant’s endogenous hormones will change to activate the mechanisms of plant stress resistance. Abscisic acid (ABA), salicylic acid (SA), and jasmonic acid (JA) can improve the tolerance of plants to heat stress [18–20]. Heat stress can lead to changes in plant secondary metabolites that act to reduce stress damage. The levels of tocopherol, flavonoids, phenylpropane and ascorbic acid precursors in the seeds of heat-resistant soybean genotypes were higher after heat stress [21]. The active flavonoid glycosides in *Clematis lasiandra* provide effective anti-tobacco mosaic virus (TMV) activity through the TMV-CP target [22]. Heat stress leads to upregulation of the oxylipin biosynthetic process and proline biosynthetic process in *Agrostis stolonifera* [23]. Activation of phenolic biosynthesis and inhibition of its oxidation promote the accumulation of phenols in plants, helping them to cope with heat shock stress [24].

Many transcription factors in plants are significantly upregulated or downregulated, and their expressions change the physiological and biochemical responses in plants to heat stress. The plant heat stress transcription factor family (HSFs) includes the most important transcription factors in plant responses to heat stress. They can induce the synthesis of heat shock proteins (HSPs) in plants to improve their heat tolerance [25–27]. *AtHsfAs* were the key transcription factors for *Arabidopsis thaliana* to increase its heat tolerance [28]. In the early phase of heat stress, *AtHsfA1a* and *AtHsfA1b* could upregulate *HPSs* and other related genes, and protect plants from cytotoxicity through the expression of related genes involved in osmotic regulation [29]. Silenced *AtHsfA2* expression in *Arabidopsis thaliana* resulted the downregulation of the *HSPs* expression, and *AtHsfA2* could protect plants against heat stress induced oxidative damage, organelle dysfunction and subsequent cell death [30].

The regulation mechanisms of transcription factors such as *MADS*, *WRKY*, *MYB*, *bHLH*, and other genes responding to heat stress signals have been studied [31–33]. *OsMADS87* is a heat-sensitive gene regulating the seed size of rice, and it has the potential to improve rice heat tolerance [34]. *AtWRKY39* could promote plant responses to heat stress through SA and JA mediated signaling pathways [35]. The *Arabidopsis pif4* mutant was early flowering but did not show rapid extension of plant axes and leaf hyponasty under heat stress. This showed that *PIF4* was an important component in heat stress response [36].

Transcriptome sequencing provides important information on gene expression patterns, functional genes, and regulatory mechanisms involved in plant abiotic stress [37, 38]. In *OsMYB55* transgenic maize, a significant number of genes involved in responses to abiotic stresses, such as high temperature, dehydration, and oxidative stress, were upregulated [39]. Several potential genes associated with heat stress were isolated after transcriptional analysis of heat-sensitive and heat-resistant varieties of Chieh-qua. Among these, several genes of *HSP*, cytochrome P450, and *bHLH* transcription factor were specifically induced [40]. In the thermotolerant rice germplasm (SDWG005), a total of 3559 differentially expressed genes (DEGs) were identified by transcriptomic analysis. The agmatine-coumarin-acyltransferase gene *OsACT* in different germplasm may be involved in the heat resistance of SDWG005 [41].

*Clematis lanuginose* was introduced to Europe in the nineteenth century and is the parent of many early varieties of *Clematis*. *Clematis crassifolia,* which is an evergreen *Clematis* with many flowers in winter, is sensitive to high temperatures in summer. In this study we used transcriptome analysis to compare different heat stress periods on *C. lanuginose* and *C. crassifolia*. We compared and analyzed the DEGs under heat stress and observed response differences between *C. lanuginose* and *C. crassifolia*. while also screening potential heat stress response genes to cultivate *Clematis* varieties with strong heat resistance.

## Results

### Effect of heat stress on plant growth

**Fig. 1:**
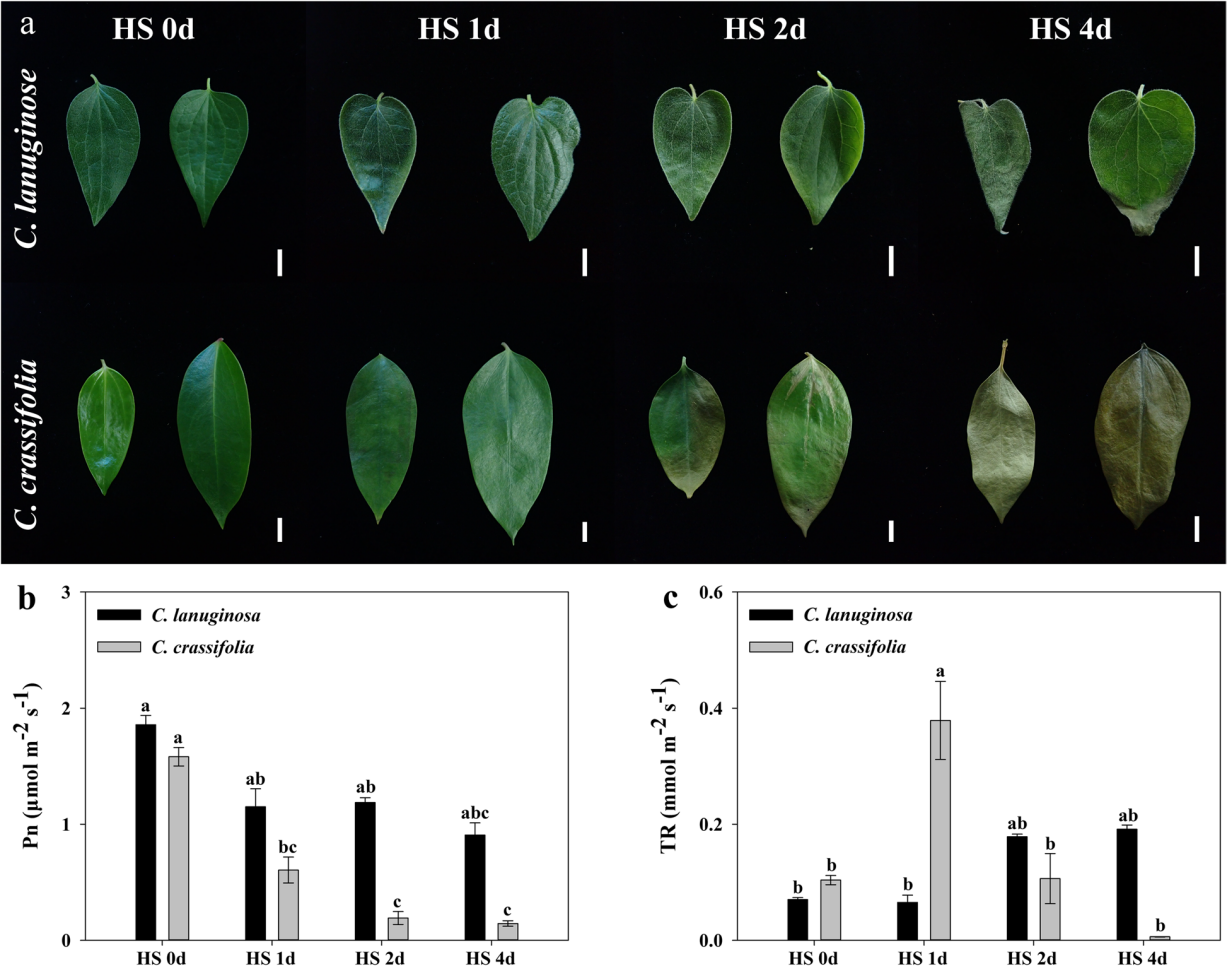
Effect of heat stress on the morphology and photosynthetic parameters of *C. lanuginose* and* C. crassifolia*. **a** The phenotype of *C. lanuginose* and *C. crassifolia* leaves; **b** Net photosynthetic rate (Pn); **c** Transpiration rate (TR). Different letters indicate significant differences based on two-way ANOVA followed by Tukey’s multiple comparison (*P* ≤ 0.05)

*C. lanuginose* had leaf tip wilting and withered new leaves 4 d after heat stress. *C. crassifolia* leaves turned yellow and wilted 2 d after heat stress (Fig. 1a). The net photosynthetic rate of *C. lanuginose* decreased by 51.21% at d 4 under heat stress, while the net photosynthetic rate of *C. crassifolia* decreased by 61.71% at d 1 and 90.87% at d 4 (Fig. 1b). The transpiration rate of *C. lanuginose* increased significantly after 2 d of heat stress, while the transpiration rate of *C. crassifolia* increased 265.14% at 1 d and decreased 94.25% at d 4 of heat stress (Fig. 1c).

### Effect of heat stress on antioxidant enzyme activities and H_2_O_2_ level

**Fig. 2:**
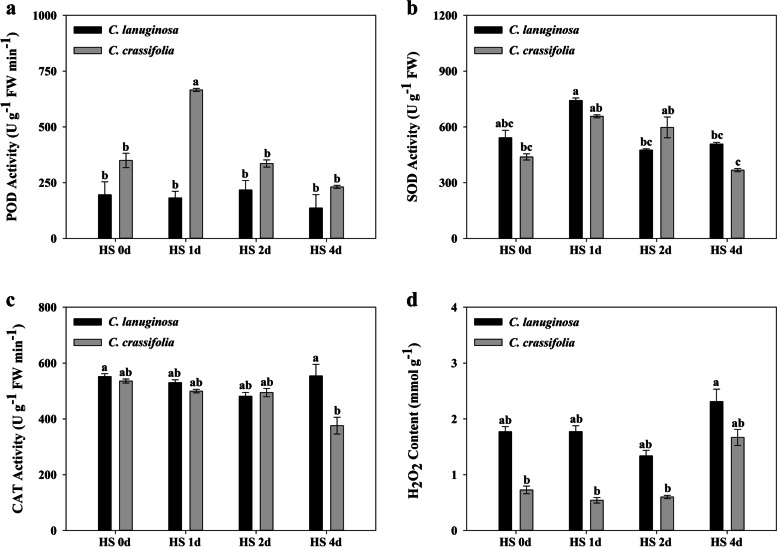
Effect of heat stress on the POD, SOD, CAT activity and H_2_O_2_ levels of *C. lanuginosa* and *C. crassifolia*. **a** POD activity;** b** SOD activity; **c** CAT activity; **d** H_2_O_2_ level. Different letters indicate significant differences based on two-way ANOVA, followed by Tukey’s multiple comparison (*P* ≤ 0.05)

The POD activity of *C. crassifolia* increased by 90.40% at d 1, and there was no significant difference in *C. lanuginose* during the heat stress (Fig. 2a). The SOD activity of *C. lanuginose* and *C. crassifolia* increased by 37.04% and 49.88% at d 1, respectively, and then gradually decreased (Fig. 2b). CAT activity of *C. crassifolia* was decreased by 29.87% at d 4 (Fig. 2c). The H_2_O_2_ levels of *C. lanuginose* and *C. crassifolia* increased by 30.63% and 109.62%, respectively, at d 4 (Fig. 2d).

### Transcriptome analyses of *C. lanuginosa* and *C. crassifolia* under heat stress conditions

The leaf tissues of *C. lanuginosa* and *C. crassifolia* were collected from HS d 0, HS d 1, HS d 2, and HS d 4, and from them we isolated total RNA and constructed eight cDNA libraries for transcriptome sequencing. The average number of clean reads was 70,390,475 and the average GC content was 46.76% in *C. lanuginosa*. In *C. crassifolia*, the average number of clean reads and GC content were 70,715,132 and 44.74%, respectively (Table 1). All the clean reads of cDNA libraries were assembled with the de-novo assembly method because *Clematis* does not have a reference genome sequence. A total of 540,495 transcripts and 395,844 unigenes were obtained after splicing (Table S1).

**Table 1:** Summary of transcriptome sequencing data of *C. lanuginosa* and *C. crassifolia*

Species	Treatments	Total reads	Bases (bp)	Q20 (%)	Q30 (%)	GC (%)	Clean reads	Clean data (bp)	Clean reads (%)	Clean data (%)
*C. lanuginosa*	HS 0d	73,738,930	11,060,839,500	95.88	90.26	46.72	72,651,440	10,710,041,936	98.52	96.82
	HS 1d	69,988,838	10,498,325,700	95.64	89.85	46.33	68,916,560	10,155,437,450	98.46	96.73
	HS 2d	71,613,838	10,742,075,700	95.30	89.16	46.93	70,442,394	10,388,088,990	98.36	96.70
	HS 4d	70,853,902	10,628,085,300	95.64	89.84	47.05	69,551,504	10,207,409,516	98.16	96.04
*C. crassifolia*	HS 0d	72,627,878	10,894,181,700	98.19	95.24	44.68	71,797,810	10,553,229,142	98.85	96.87
	HS 1d	70,425,668	10,563,850,200	97.94	94.73	43.99	69,538,524	10,244,327,340	98.74	96.97
	HS 2d	69,231,504	10,384,725,600	97.95	94.75	45.18	68,335,950	10,054,571,138	98.70	96.82
	HS 4d	74,139,482	11,120,922,300	98.12	95.07	45.12	73,188,242	10,744,646,388	98.71	96.61

### Annotation of the transcriptome

**Fig. 3:**
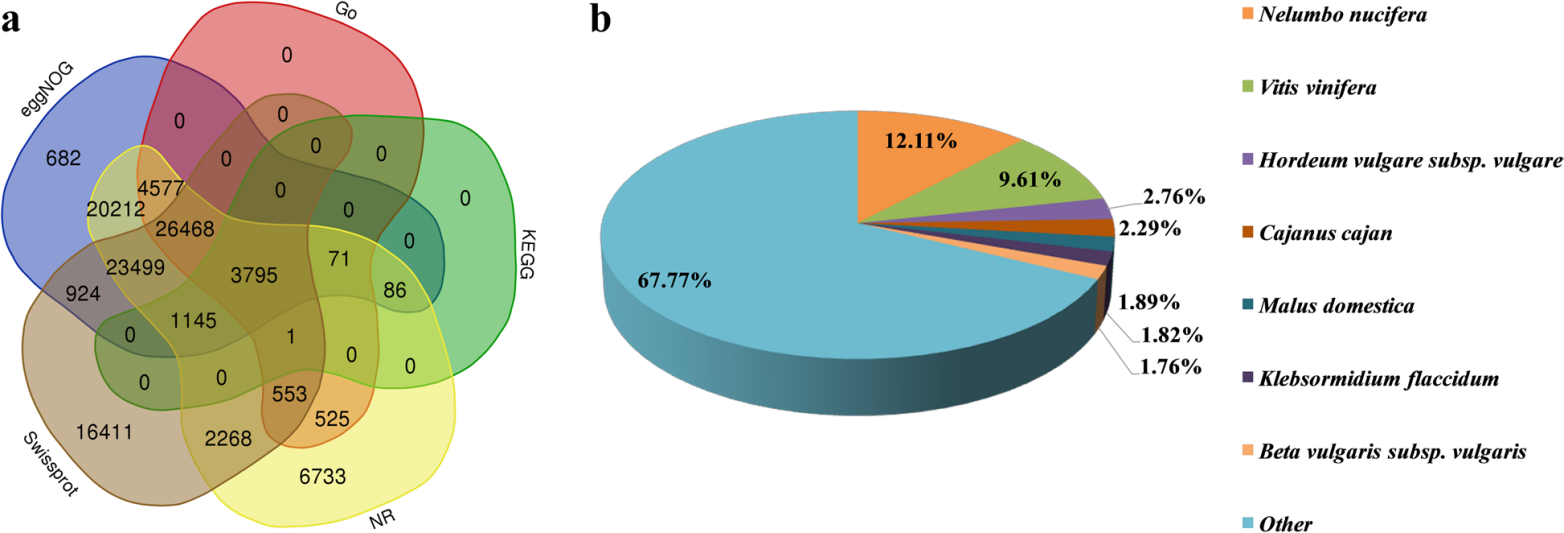
Annotation of the transcriptome. **a** Gene function annotations in five databases (NR, Swiss-Prot, GO, KEGG, eggNOG); **b** Homologous species distribution of the annotated in the NR database

The gene function annotation results showed that a total of 89,933 genes were annotated to the NCBI non-redundant (NR) database, 35,990 genes were annotated to the Gene Ontology (GO) database, 5,098 genes were annotated to the Kyoto Encyclopedia of Genes and Genomes (KEGG) database, 81,459 genes were annotated to the evolutionary genealogy of genes: Non-supervised Orthologous Groups (eggNOG) database, and 75,064 genes were annotated to the Swiss-Prot database. Of these, 3,795 genes were annotated in all databases (Fig. 3a). According to the GO annotation analysis, there were 18,943, 17,841, 1,965, 1,227, and 304 genes with the molecular functions of catalytic activity, binding, transporter activity, structural molecule activity, and antioxidant activity, respectively (Fig. S1).

According to sequence homology alignment results, 10,889 (12.11%) genes were homologous to *Nelumbo nucifera*; 8644 (9.61%) genes had significant hits for *Vitis vinifera*, followed by *Hordeum vulgare* subsp. *vulgare* (2481, 2.76%), *Cajanus cajan* (2055, 2.29%), *Malus domestica* (1699, 1.89%), *Klebsormidium flaccidum* (1636, 1.82%) and *Beta vulgaris* subsp. *vulgaris* (1585, 1.76%). A total of 60, 943 genes were homologous to other species (Fig. 3b).

### Gene expression profiles of *C. lanuginosa* and *C. crassifolia* under heat stress

**Fig. 4:**
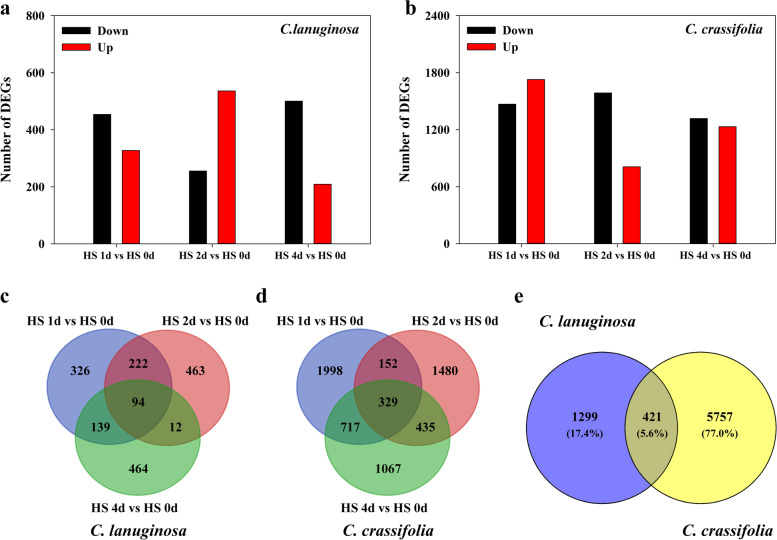
The number of up and down regulated differentially expressed genes (DEGs) and Venn diagram in *C. lanuginosa* and *C. crassifolia*. **a** The DEG number of C. lanuginosa; **b** The DEG number of *C. crassifolia*; **c** Venn diagrams for DEGs in the three comparison groups of *C. lanuginosa*; **d** Venn diagrams for DEGs in the three comparison groups of *C. crassifolia*; **e** Venn diagrams for DEGs between *C. lanuginosa* and *C. crassifolia*

Many genes were differentially expressed in *C. lanuginosa* and *C. crassifolia* under heat stress. After 1, 2, and 4 d of heat stress treatment, 327, 536 and 209 genes were upregulated and 454, 255 and 500 genes were downregulated, respectively, in *C. lanuginosa* (Fig. 4a). In *C. crassifolia*, after 1 d of heat stress, 1727 genes were upregulated and 1469 genes were downregulated. After 2 and 4 d of heat stress, there were 810 and 1231 genes upregulated, 1586 and 1317 genes downregulated, respectively (Fig. 4b).

Under the heat stress treatment, 94 genes were significantly differentially expressed at different times (HS d 1, HS d 2, and HS d 4) in *C. lanuginosa*, while 329 genes in *C. crassifolia* (Figs. 4c, d**)**. Among all the differentially expressed genes, 421 (5.6%) genes were significantly differentially expressed in both *C. lanuginosa* and *C. crassifolia*, 1299 (17.4%) genes were specifically expressed in *C. lanuginose* and 5757 (77.0%) genes were uniquely expressed in *C. crassifolia* (Fig. 4e).

### GO annotation of DEGs in *C. lanuginosa* and *C. crassifolia* under heat stress

Go enrichment analysis was performed on the DEGs of *C. lanuginosa* and *C. crassifolia*. In the comparison groups of HS d 1 vs. HS d 0, 314 (40.20%) and 809 (25.31%) DEGs were annotated into the GO terms in *C. lanuginose* and *C. crassifolia*, respectively. For HS d 2 vs. HS d 0 comparison groups, 173 (21.87%) and 628 (26.21%) DEGs were annotated in *C. lanuginose* and *C. crassifolia*, respectively*.* In the groups of HS d 4 vs. HS d 0, 320 (45.13%) and 625 (24.53%) DEGs in *C. lanuginose* and *C. crassifolia* were annotated, respectively (Fig. S2).

**Fig. 5:**
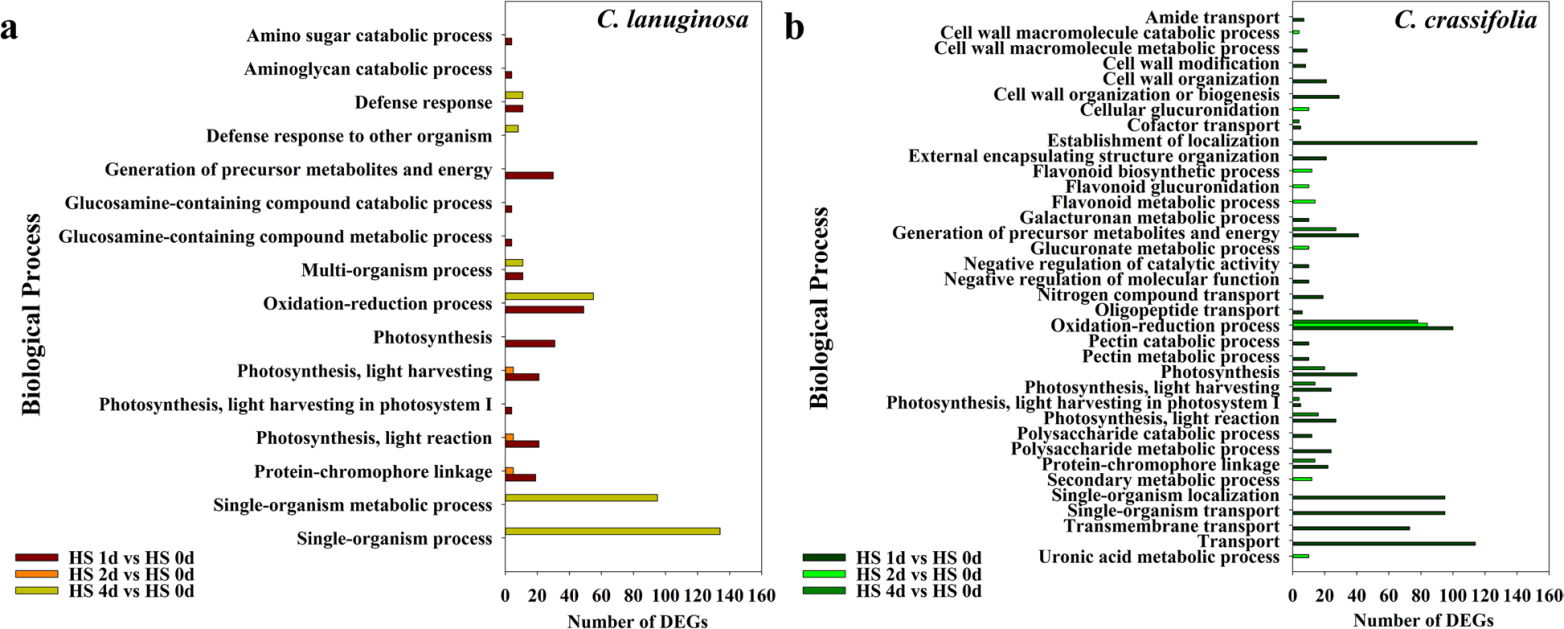
Enriched GO terms (Biological process) (*P* < 0.05) of DEGs. **a** *C. lanuginosa*; **b** *C. crassifolia*

In *C. lanuginosa,* DEGs were mainly enriched in the terms involved in biological processes, such as single-organism process, oxidation–reduction process, and single-organism metabolic process. The oxidation reduction process, as well as the establishment of the localization and transport of DEGs, were observed in *C. crassifolia* (Fig. 5).

**Fig. 6:**
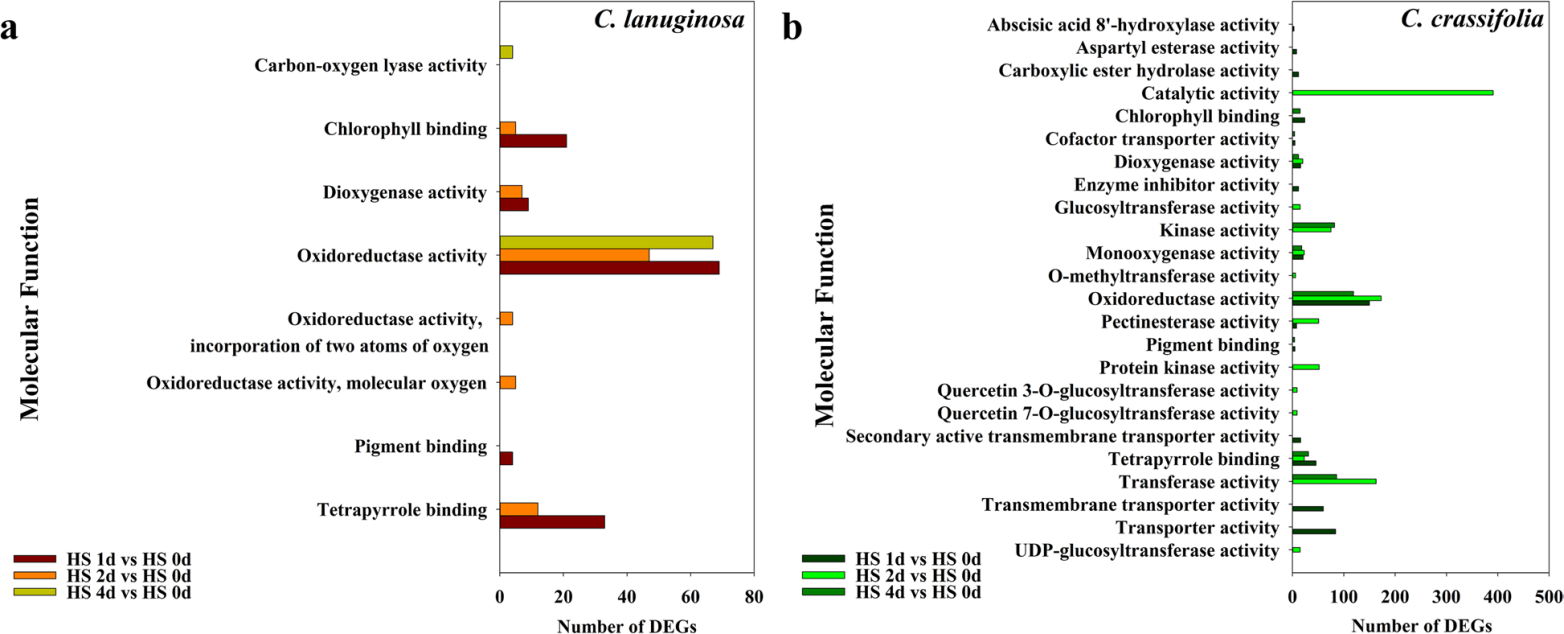
Enriched GO terms (Molecular function) (*P* < 0.05) of DEGs. **a** *C. lanuginosa*; **b** *C. crassifolia*

For molecular functions, the genes were mostly enriched in oxidoreductase activity, tetrapyrrole binding, chlorophyll binding, and dioxygenase activity in *C. lanuginosa*. They were mainly enriched in oxidoreductase activity, catalytic activity, transferase activity, and kinase activity in *C. crassifolia* (Fig. 6). Within the cellular component category, the DEGs were commonly enriched in the terms of membrane, chloroplast, and plastid in *C. lanuginosa*. In *C. crassifolia,* DEGs were mainly concentrated in the membrane, intracellular membrane-bounded organelle, membrane-bound organelle, and mitochondrion (Fig. S3).

### KEGG annotation and unique DEGs in *C. lanuginosa* and *C. crassifolia* under heat stress

**Fig. 7:**
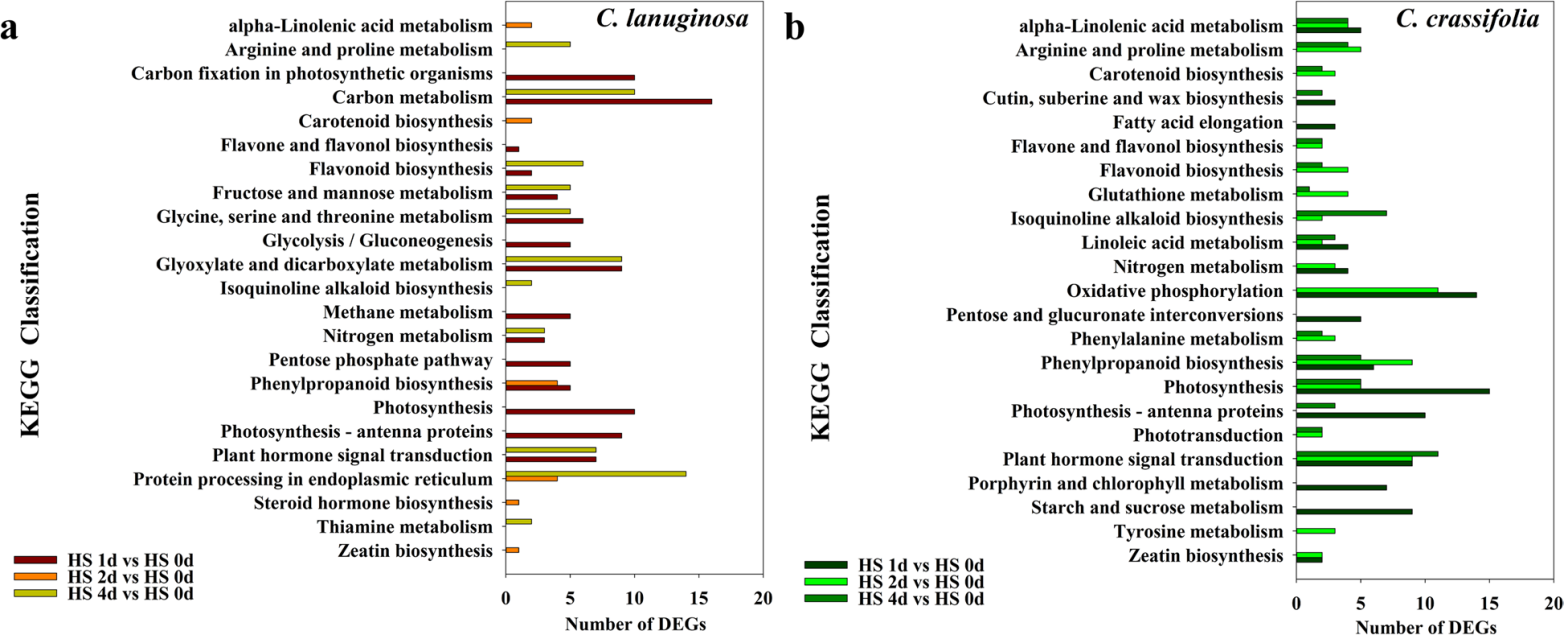
KEGG enrichment analysis of DEGs (P < 0:05) in *C. lanuginosa* and *C. crassifolia*. **a** *C. lanuginosa*; **b** *C. crassifolia*

In *C. lanuginosa*, all the DEGs of the three comparison groups HS d 1 vs. HS d 0, HS d 2 vs. HS d 0, and HS d 4 vs. HS d 0 were annotated into 57, 35, and 86 KEGG pathways, respectively, and more DEGs were significantly distributed in 23 pathways including carbon metabolism, carbon fixation in photosynthetic organisms, glyoxylate and dicarboxylate metabolism. Under the condition of heat stress, there were 116, 124 and 128 KEGG pathways annotated by DEGS in *C. crassifolia*, respectively, significantly focusing on 23 pathways, such as oxidative phosphorylation, photosynthesis, plant hormone signal transduction, and phenylpropanoid biosynthesis (Fig. 7). We analyzed all the DEGs in *C. lanuginosa* and *C. crassifolia*, and 34 unique genes related to heat stress were observed in *C. lanuginosa* (Table S2). A total of 29 DEGs related to heat stress were independently expressed in *C. crassifolia* (Table S3).

### Identification of the heat shock protein, antioxidant enzyme, photosynthetic-related genes and transcription factors in *C. lanuginosa* and *C. crassifolia* under heat stess

**Fig. 8:**
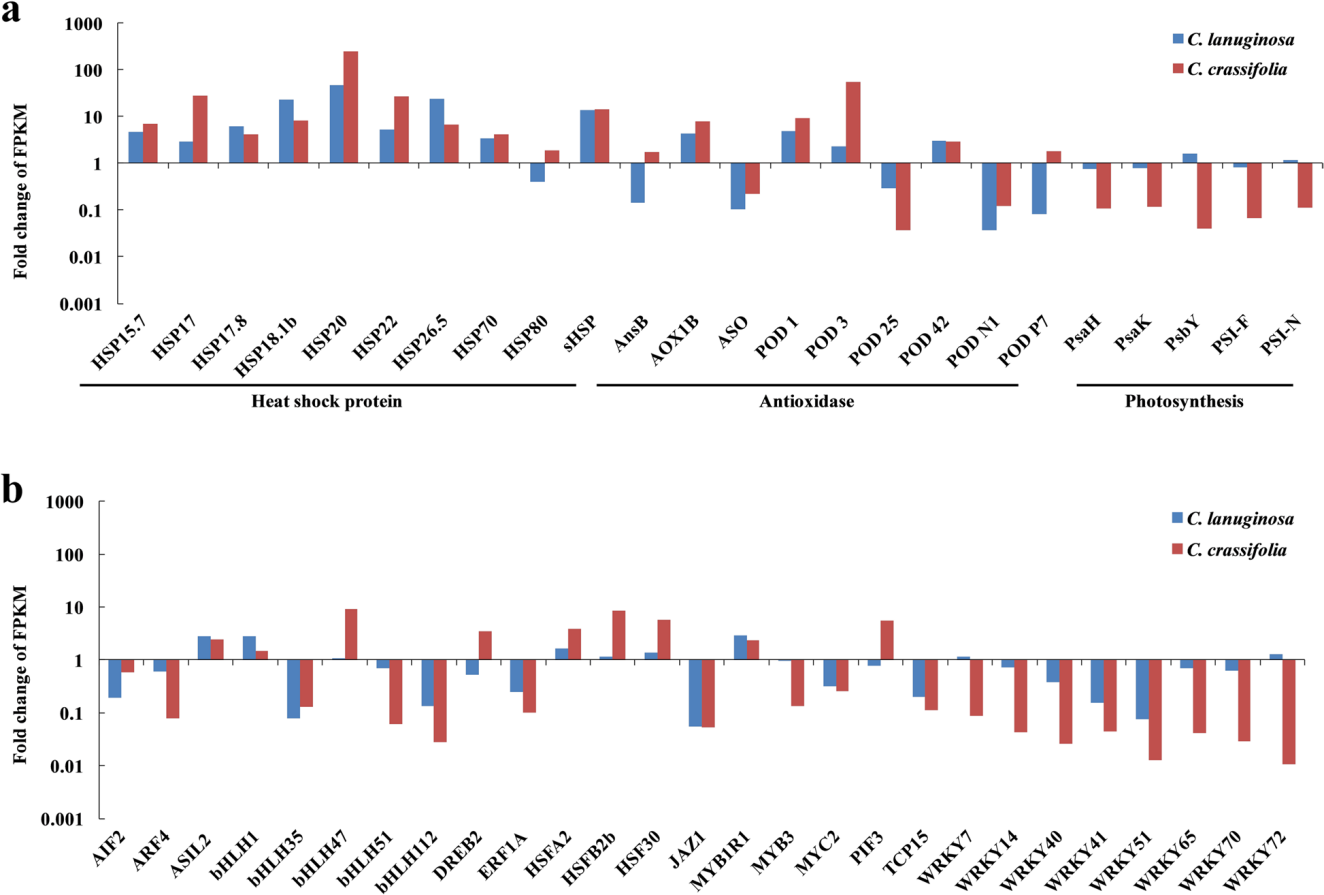
Expression of transcription factors (TFs), heat shock protein, antioxidant enzyme, and photosynthesis-related genes of HS d 4 vs. HS d 0 in *C. lanuginosa* and *C. crassifolia* by RNA-seq dataset. a Gene expression of heat shock proteins, antioxidant enzyme and photosynthetic-related genes; b Gene expression of TFs

To evaluate the potential regulation in *C. lanuginosa* and *C. crassifolia* under heat stress, we searched for transcription factors, heat shock protein, antioxidant enzyme and photosynthetic genes from the RNA-seq dataset. There were eight heat shock proteins, nine antioxidant enzyme genes and four photosynthesis-related genes showing the same expression trend, while *HSP80* (*c196872_g2*), *PODP7* (*c204229_g1*) and *PsbY* (*c200811_g3*) showed different expression trends in *C. lanuginosa* and *C. crassifolia* (Fig. 8a). In 27 candidate transcription factors, nine transcription factors showed a significant increase or reduced expression, including *ASIL2* (*c195867_g1*), *bHLH112* (*c203571_g1)*, *JAZ1* (*c194555_g1*), *MYBR1* (*c204139_g1*), *MYC2* (*c208293_g1*), *TCP15* (*c195225_g2*), *WRKY40* (*c189120_g1*), *WRKY41* (*c200654_g1*), and *WRKY51* (*c187717_g1*). Four transcription factors including *DREB2* (*c182557_g1*), *PIF3* (*c209598_g2*), *WRKY7* (*c206794_g2*), and *WRKY72* (*c203219_g1*) showed opposite expression trends in the two *Clematis* species (Fig. 8b).

**Fig. 9:**
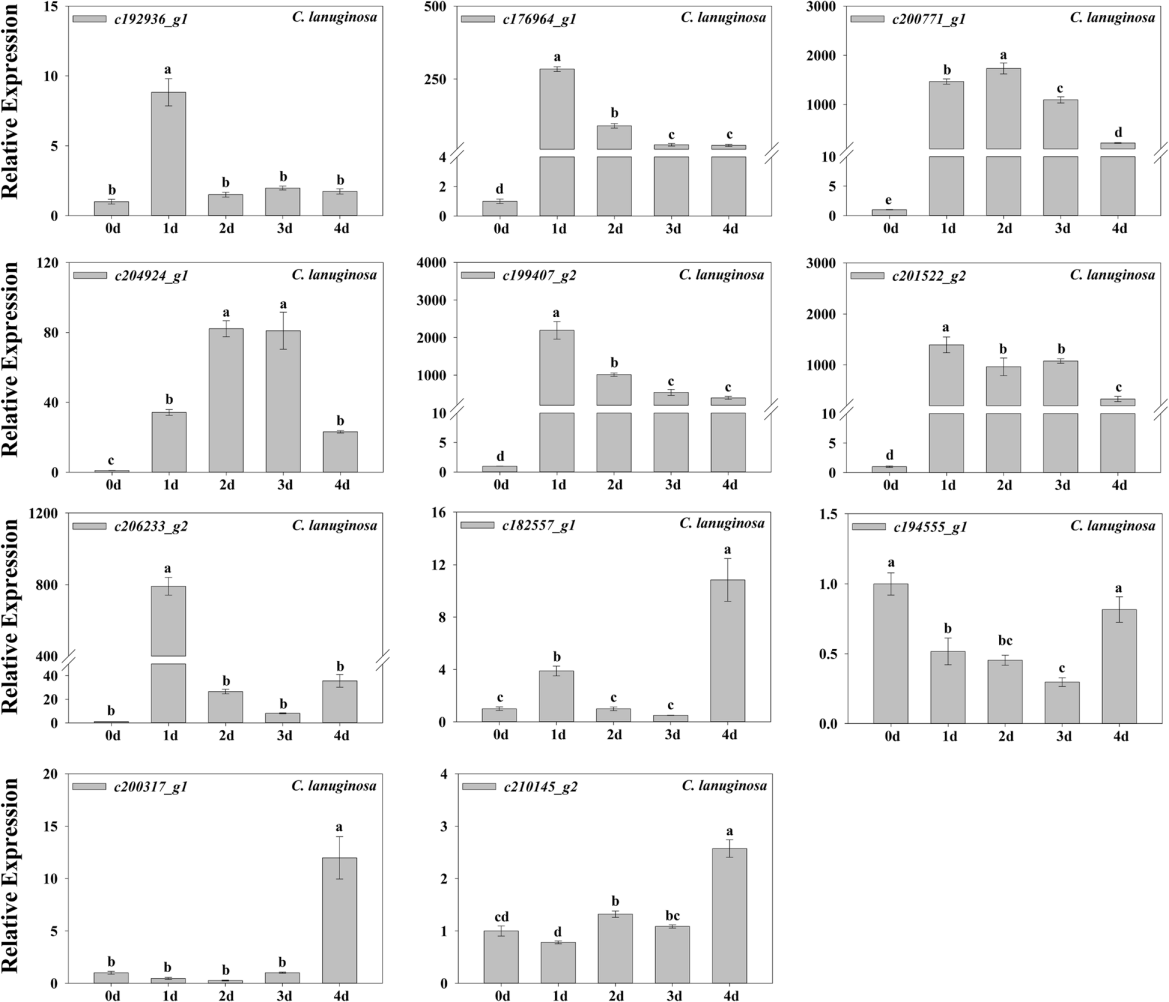
Fold change of gene expression in *C. crassifolia*. Bars indicate SE (*n* = 3). Different letters indicate significant differences based on one-way ANOVA followed by Tukey’s multiple comparison (*P* ≤ 0.05)

We selected some DEGs annotated as HSPS, antioxidant enzymes and transcription factors for q-PCR analysis. The results showed that the expression of six HSPS genes were upregulated in *C. lanuginosa* and *C. crassifolia*, but the expression trends were different. *HSP17.8* (*c176964_g1*), *HSP26.5* (*c200771_g1*), *HSP70* (*c204924_g1*), *HSP18.1* (*c199407_g2*) and *HSP20* (*c201522_g2*) were significantly upregulated from d 1 to the end of heat stress, and *HSP17* (*c192936_g1*) and *HSFA2* (*c206233_g2*) were significantly upregulated on d 1. *DREB2* (*c182557_g1*), *POD1* (*c200317_g1*) and *POD3* (*c210145_g2*) were significantly upregulated at 4 d of heat stress. *JAZ1* (*c194555_g1*) was significantly downregulated from d 1 to d 3, and upregulated at d 4 (Fig. 9).

**Fig. 10:**
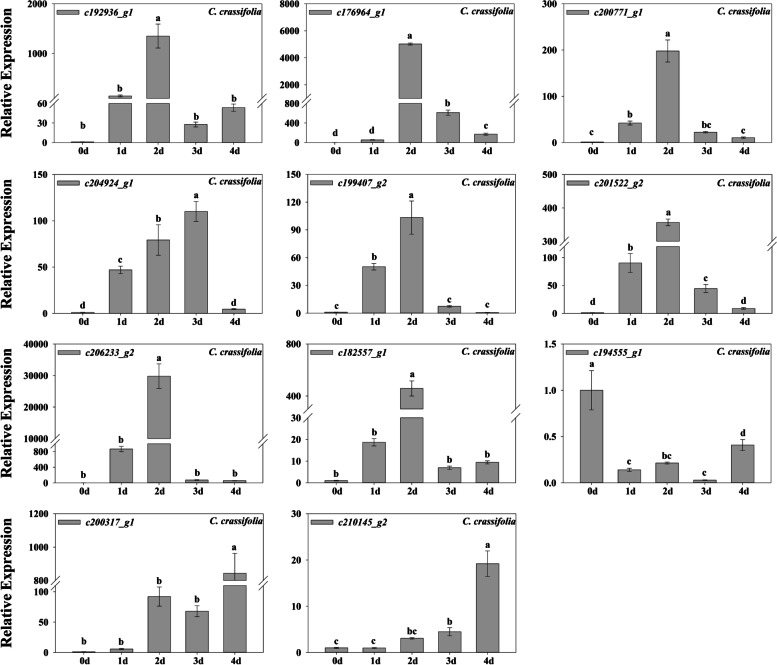
Fold change of gene expression in *C. crassifolia*. Bars indicate SE (*n* = 3). Different letters indicate significant differences based on one-way ANOVA followed by Tukey’s multiple comparison (*P* ≤ 0.05)

In *C. crassifolia*, *HSP17* (*c192936_g1*), *HSP17.8* (*c176964_g1*), *HSP26.5* (*c200771_g1*), *HSFA2* (*c206233_g2*), *DREB2* (*c182557_g1*) all increased significantly after 2 d of heat stress, and *HSP70* (*c204924_g1*), *HSP18.1* (*c199407_g2*), *HSP20* (*c201522_g2*) were significantly upregulated on the first day. *POD1* (*c200317_g1*) and *POD3* (*c210145_g2*) were significantly upregulated under heat stress (Fig. 10).

## Discussion

*C. lanuginosa* and *C. crassifolia* showed distinct leaf damage phenotypes under heat stress (Fig. 1). Transcriptome data analysis indicated 1720 differentially expressed genes in *C. lanuginosa*, of which 1299 (75.52%) were specifically and significantly expressed in the leaf. There were 6178 DEGs, among which 5757 (93.19%) genes were significantly expressed only in *C. crassifolia*. These results indicate that the gene expression in *C. crassifolia* was more sensitive to heat stress (Fig. 4).

Photosynthesis is a plant physiological process sensitive to heat. Heat stress has negative effects on photosynthesis by destroying the electron transport chain, carbon metabolism and the Photosystem II (PSII) system oxygen releasing complex [42, 43]. Analysis of the net photosynthetic rate of *C. lanuginosa* and *C. crassifolia* showed that the net photosynthetic rate of *C. crassifolia* continued to decrease significantly under heat stress (Fig. 1b). During the initial stage, 2 d after heat stress in the GO enrichment analysis, photosynthesis light harvesting and the light reaction process were inhibited in *C. lanuginosa* and *C. crassifolia*. In the later stage of heat stress, the plants produced precursor metabolites and energy to regulate light capture and other functional genes and maintain photosynthesis (Figs. 5 and 6). Photosystem I (PSI) is a multiprotein complex composed of two large subunits of 82 kD and several small subunits of less than 20 kD in the plant chloroplast thylakoid membrane, which mediates the light-driven electron transfer from plastocyanin to Fd [44]. The genes encoding these subunits include PsaA—PsaL [45]. In the later period of heat stress, *PsaH*, *PsaK*, *PsbY*, *PSI—F*, and *PSI—N* genes were downregulated more in *C. crassifolia*, indicating that the inhibitory effect of heat stress on PSI in *C. crassifolia* was greater than that in *C. lanuginose* (Fig. 8).

Plants will accumulate ROS such as ^1^O_2_, O^2−^, H_2_O_2_, and OH^−^ under heat stress, which will impair chloroplast and mitochondrial functions, subject the plant cells to oxidative damage, including lipid peroxidation, protein oxidation, and DNA damage. H_2_O_2_ is one of ROSs which is closely related to oxidative stress. It is derived from superoxide anion disproportionation, and the product of H_2_O_2_ has strong oxidation ability [46, 47]. During evolution, plants have developed an enzymatic antioxidant system to remove excess ROS, which is dominated by ascorbate peroxidase (APX), SOD, POD, and CAT. The antioxidant enzyme activities are positively correlated with the heat tolerance of plants [48–50]. *SlMAPK3* was a negative regulator of thermotolerance in *Solanum lycopersicum*, *slmapk3* mutants have higher activities and transcript levels of POD, SOD, CAT, and APX than wild type plants [51]. There was no significant change in the content of H_2_O_2_ in *C. lanuginosa*, which might be related to the maintenance of antioxidant enzyme activity. In *C. crassifolia*, the increase of SOD and POD activities on d 1 and d 2 maintained ROS balance at the early stage of heat stress (Fig. 2). Gene enrichment in the oxidation reduction process improved the ability of *C. lanuginosa* and *C. crassifolia* to eliminate reactive oxygen species so that the plants could maintain the short-term balance of ROS. The biological processes related to establishment of localization, transport, and oxidation reduction process were significantly enriched in *C. crassifolia* at d 1 under heat stress (Figs. 5 and 6). However, the activities of CAT, POD, and SOD of *C. crassifolia* relatively decreased after 4 d, and the H_2_O_2_ content increased significantly at d 4, indicating that the protective enzyme system had a strong time dependence in response to heat stress in *C. crassifolia* (Fig. 2d).

Flavonoids have a variety of biological functions including antioxidant, antiviral, auxin transport, and antimicrobial [52, 53]. The increase in flavonoid substances in some plant species can enhance their ability to resist biological and abiotic stress [54]. Heat stress reduced the fertilization success of *Ipomoea purpurea*, and flavonoids could ameliorate the adverse effects of heat stress on fertilization and early seed maturation [55]. In *C. lasiandra*, three flavonoids including kaempferol 3-*O*-*α*-L-rhamnopyranoside, ^34^isovitexin 6''-*O*-*E*-*ρ*-coumarate, and ^35^quercetin 3-O-*β*-D-glucopyranuronide showed higher anti-TMV active compared with ningnanmycin, especially ^34^isovitexin 6''-*O*-*E*-*ρ*-coumarate could directly fracture TMV particles into small fragments combining with the fusion phenomena. The DEGs of *C. lanuginosa* and *C. crassifolia* were annotated in the KEGG classification of flavone and flavonol biosynthesis and flavonoid biosynthesis, suggesting that flavonoids played important role in the response of heat stress (Fig. 7).

Amino acids are involved in protein synthesis. Plants can promote the synthesis of proteins involved in photosynthesis, enzymatic antioxidant system and stress signals by accumulating amino acids under heat stress. These can also protect the lipids in thylakoid membranes from damage [56, 57]. The accumulation of glyoxylic acid can affect the expression of modified proteins or stress-related genes in plants under high light and heat stress [58]. Thiamine plays an important role in metabolic pathways such as glycolysis, nicotinamide adenine dinucleotide phosphate (NADPH) and adenosine-triphosphate (ATP) synthesis, and is activated as an enzyme cofactor in plants responding to abiotic stress [23, 59, 60]. In the KEGG analysis of *C. lanuginosa*, genes related to glycine, serine, and threonine metabolism, glyoxylate and dicarboxylate metabolism, protein processing in the endoplasmic reticulum and thiamine metabolism classificantions were expressed differentially compared to *C. crassifolia* (Fig. 7a). This indicates that the accumulation of amino acids and the activation of thiamine metabolic pathways may be important in the response of *C. lanuginosa* to heat stress. A previous study confirmed that heat stress can promote the accumulation of arginine and proline content in *C. crassifolia* [61]. The differentially expressed genes in *C. crassifolia* were specifically upregulated in phenylalanine metabolism, arginine/proline metabolism and the flavonoid biosynthesis classificantions. These may be important pathways for *C. crassifolia* to respond to heat stress (Fig. 7b). Heat shock proteins are highly conserved proteins in plants that have anti-stress effects. Based on their molecular size, they are mainly classified into HSP110, HSP90, HSP70, HSP60 and sHSPs [62, 63]. Under heat stress, heat shock proteins can bind to other proteins as molecular chaperones to maintain protein homeostasis, repair denatured proteins, and assist in protein transport. After heat stress treatment, the significantly upregulated heat shock proteins in *C. lanuginosa* and *C. crassifolia* were mainly concentrated in *sHSPs*, including the six genes *HSP17*, *HSP17.8*, *HSP18.1*, *HSP20*, *HSP26.5*, and *HSP70* (Figs. 8a, 9 and 10). This finding was similar to the upregulation of small heat shock proteins in the transcriptomes of *C. apiifolia* under heat stress [64]. The chaperone activity of small heat shock proteins involves passively mediating the synthesis and release of substrates without using ATP [65]. Also, the small heat shock proteins have a cross protection function and can adjust the membrane fluidity, interact with thylakoid membrane and reduce plasma membrane fluidity to maintain cell homeostasis [66, 67]. In the annotation analysis of DEGs in *C. lanuginosa* and *C. crassifolia*, many DEGs were involved in the composition of the cell membrane, chloroplast thylakoid membrane, thylakoid membrane and plastid thylakoid membrane. These results indicated that the upregulated expression of these six sHSPs genes may protect the stability of the *Clematis* membranous system.

Transcription factors are important in the signal transduction process of plants in response to stress [68]. The increase or decrease in the expression of transcription factors can regulate downstream gene expression while also transmitting and amplifying the stress signals. *HSF*, *WRKY*, *MADS*, *bZIP*, *MYB*, *bHLH*, *AP2/EREBP*, *NAC*, and other transcription factors genes are closely related to plant abiotic stress responses [69, 70]. In Arabidopsis, *hsfa2* mutants were more sensitive to heat stress, and silencing *AtHsfA2* resulted in downregulation of HSPs gene expression [71, 72]. Similarly, we observed that *HSFA2* (*c206233_g2*) was significantly upregulated in the early and late stages of heat stress in *C. crassifolia* (Figs. 6b and 10), suggesting that *HSFA2* may play an important role in the heat response of *C. crassifolia*. *CvHSF30-2* was a transcription factor induced by heat stress in *Clematis vitalba*, which improved the heat tolerance of *C. vitalba* by increasing the expression of HSPs [73]. Upregulation of *HSF30* (*c194517_g1*) was also observed in *C. lanuginosa* and *C. crassifolia* (Fig. 8; Fig. S5). JAZ is a negative regulator of the jasmonic acid (JA) signal response pathway. Exogenous application of meJA can enhance the heat tolerance of wheat, while *HSFA1b* can regulate the expression of the JA synthesis gene *AtOPR3* in *Arabidopsis thaliana* by combining heat shock elements (HSE) [74]. *JAZ1* (c194555_g1) was significantly downregulated during heat stress in *C. lanuginosa* and *C. crassifolia* (Figs. 8b, 9 and 10), suggesting that the *JAZ1* transcription factor may regulate jasmonic acid signaling pathways to adapt to heat stress. *DREB2* plays an important role in heat stress, and its over-expression can improve plant heat tolerance [75]. *WRKY72* in rice can be upregulated by heat stress, and may be involved in a variety of plant biological processes [76]. Soybean studies indicate that PIF3 may be a potential target gene for regulating weed tolerance in soybean [77]. The *c182557_g1* gene was annotated as encoding *DREB2*. The *c182557_g1* was upregulated in *C. lanuginosa* and *C. crassifolia*, but its expression patterns were different (Fig. 9; Fig. 10). *PIF3* (*c209598_g2*) and *WRKY72* (*c203219_g1*) were upregulated in *C. lanuginosa* and *C. crassifolia*, respectively (Fig. 8b). However, the regulatory roles of these genes in response to heat stress in *Clematis* remains to be verified. This study provides a reference for further analyzing the molecular regulatory mechanism of *Clematis* in response to heat stress and the breeding of *Clematis* cultivars with increased heat tolerance.

## Conclusions

In this study, the transcriptomes of *C. lanuginosa* and *C. crassifolia* were assembled. A total of 1,720 and 6,178 DEGs were identified from *C. lanuginosa* and *C. crassifolia*, respectively. DEGs enrichment of metabolic pathways and gene expression analysis showed that glycine/serine/threonine metabolism, glyoxylic metabolism and thiamine metabolism were important pathways in the response to heat stress in *C. lanuginosa.* Flavonoid metabolism, phenylalanine metabolism, and arginine/proline metabolism were the key pathways in *C. crassifolia.* Several candidate genes that may be involved in the response of *C. lanuginosa* and *C. crassifolia* to heat stress were identified, and these indicated that *C. lanuginosa* and *C. crassifolia* have different response strategies to heat stress.

## Methods

### Plant materials and growth conditions

The *Clematis lanuginosa* Lindl*.* and *Clematis crassifolia* Benth. used in this study were provided by the Zhejiang Institute of Subtropical Crops, Wenzhou, Zhejiang Province, China. It was identified by Professor Jian Zheng. These species were preserved in National Clematis Germplasm Resource Center, Wenzhou, Zhejiang Province, China (*C. lanuginose* voucher code: W-2016–43; *C. crassifolia* voucher code: W-2016–58). *C. lanuginosa* and *C. crassifolia* plants were grown in the Zhejiang Institute of Subtropical Crops, China. Healthy, two-year-old plants were grown in a grown chamber under 25/20 °C (16:8 h (L:D) photoperiod); 65% humidity) conditions for two weeks. After two weeks of pretreatment, *C. lanuginosa* and *C. crassifolia* plants were transferred to a growth chamber for cultivation at 45/40 °C temperature and 16:8 h (L:D) photoperiod. The heat stress treatment duration was 4 d. During the treatment period, 500 ml of water was given to each plant every 2 d, to ensure sufficient soil moisture. The leaves sampled before the heat stress were labelled as “HS 0d,” and those after heat stress were labelled as HS 1d, HS 2d, and HS 4d. Experimental treatments were repeated three times. All methods, including plant experimental research, were in compliance with the relevant guidelines, regulations and legislation.

### Leaf gas exchange parameters

Healthy and fully developed leaves were randomly chosen for photosynthetic parameter measurements, using LI-6400 XT portable photosynthesis system (Li-Cor Inc., Lincoln, NE, USA), and equipped with a 6400–18 RGB LED light source. The measurements were carried out from 9:00 to 11:00 am, the photosynthetic photon flux density was 1200 μmol m^−2^ s^−1^, the CO_2_ concentration was 400 ppm, and the relative humidity was 65%.

### Determination of Superoxide Dismutase (SOD), Catalase (CAT), Peroxidase (POD) activity and H_2_O_2_ content

For peroxidase enzyme activity analysis, fresh leaves (0.1 g) were ground in liquid nitrogen and suspended in 8.0 ml solution containing 50 mM phosphate buffer (pH 7.4). The homogenate was centrifuged 15 min (10,000 rpm) at 4 °C, and then the supernatant was collected to obtain crude enzymes.

SOD activity was analyzed by measuring the inhibition rate of the enzyme to O_2_^−^ produced. SOD activity was determined at 550 nm in absorbance after 40 min of reaction at 37 °C. One-unit of SOD activity (U) was defined as the amount of enzyme that resulted in 50% inhibition of reduction of nitrite in 1 ml of reaction solution.

CAT activity was determined by the hydrolysis reaction of H_2_O_2_ with CAT, and the yellow MA-H_2_O_2_ complex was generated by adding ammonium molybdate to quickly stop the reaction. CAT activity was calculated at 405 nm. One-unit was defined as the amount of enzyme that resulted in the decomposition of 1 µmol H_2_O_2_ per second in 1.0 g fresh tissue.

POD activity was measured at 470 nm by catalyzing H_2_O_2_ based on the change of absorbance. One-unit was defined as the amount of enzyme that resulted in the change of 0.01 at 470 nm per minute by 1.0 g fresh tissues in the reaction system. Leaf tissue amounting to 0.2 g was finely ground with 25 mL acetone and homogenized at 0 °C; the content was calculated using H_2_O_2_ as the standard [78].

The H_2_O_2_ content was measured according to the method described by Patterson [79]. A 0.2 g amount of leaf tissue was finely ground finely homogenized with 25 ml of acetone at 0 °C. The H_2_O_2_ content was calculated using H_2_O_2_ as the standard.

### RNA extraction, cDNA library construction, and Illumina sequencing

Total RNA from different leaf samples of *C. lanuginosa* and *C. crassifolia* was extracted using TRIZOL reagent (Takara, Beijing, China). The concentration and purity of the total RNA was tested with an Agilent 2100 Bioanalyzer. The mRNA from total RNA was purified by the polyA structure unique to mRNA and mRNA with the polyA structure was enriched by Oligo(dT) magnetic beads. The first strand of cDNA was synthesized using 6-base random primers and reverse transcriptase using mRNA as a template. The second strand of cDNA was synthesized using the first strand of cDNA as template. The chain-specific library was established, and the quality of the library was detected by Agilent 2100 Bioanalyzer. Next Generation Sequencing (NGS) was used to perform paired-end (PE) sequencing (Illumina HiSeq X-Ten, San Diego, CA, USA; Sequencing company: Personalbio, HangZhou, China).

There was no reference genome in the transcriptome sequencing of *Clematis*, so Trinity software (r20140717) was used to splice clean reads to obtain the transcript for subsequent analysis. Trinity is a De Novo assembly software for transcriptome splicing, splicing high-quality sequences based on the DBG (De Bruijn Graph) splicing principle [80]. The longest Transcript under each gene was extracted as the representative sequence of the gene and the transcript and unigene sequences were statistically analyzed.

### Gene function annotation

NR, Swiss-Prot, eggNOG and KEGG databases (www.kegg.jp/kegg/kegg1.html) were used to annotate all the unigenes (E value < 1.0 e^−5^) [81–83]. GO annotation was performed through Blast2GO based on NR annotation results. Based on the above comparison results, protein functional annotation information of unigenes was obtained.

### Differentially expressed genes

Gene expression were analyzed using the FPKM (Fragments Per Kilobase of exon model per Million mapped reads) method. The criteria for screening DEG were p-value ≤ 0.05, false discovery rate (FDR) < 0.001, Fold Change ≥ 2, or Fold Change ≤ 0.5. Subsequently, GO and KEGG databases were used to analyze the main functions and metabolic pathways of the DEGs.

#### Quantitative real-time PCR of genes in different time under heat stress

Total RNA was extracted from leaves and the cDNA was synthesized using the Revert Aid RT Kit (Thermo Scientific, Waltham, MA, USA), Primers designed with Primer Premier 5.0 are shown in Table S5. The qRT-PCR experiment was carried out using an ABI PRISM 7500 Real-time PCR System (Applied Biosystems, Foster City, CA, USA) and AceQ qPCR SYBR Green Master Mix (Vazyme, Nanjing, Jiangsu Province, China). The PCR-PCR reaction system was as follows: 95 °C for 5 min, followed by 40 cycles of 95 °C for 15 s, and 60 °C for 30 s. Each sample was repeated three times, and the internal normalizations was *GAPDH* gene. Each primer pair was validated the specificity by melt curve analysis, and the gene expression levels were calculated by the 2^−△△Ct^ method.

### Statistical analysis

Data were analyzed by one-way or two-way ANOVA using the SPSS 10 program (SPSS Inc., Chicago, IL, USA). Different letters on the histograms between different treatments indicate a significant difference at *P* ≤ 0.05.

## Supplementary Information

**Additional file 1: Fig. S1. **Gene Ontology classification and KEGG analysis of the transcriptome.** Fig. S2. **Enriched GO terms of DEGs.** Fig. S3. **Enriched GO terms (cellular component) (P < 0.05) of DEGs.** Fig. S4. C**lustering analysis of gene expression. 

**Additional file 2: Table S1. **Statistics of splicing results.** Table S2. **Annotation of unique DEGs related to heat stress in *C. lanuginose*.** Table S3. **Annotation of unique DEGs related to heat stress in *C. crassifolia*.** Table S4. **The GenBank number of the unigenes.** Table S5. **Primer sequences for gene expression analysis. 

## Data Availability

The raw transcriptome data have been deposited at the NCBI Sequence Read Archive with accession number PRJNA702123 (https://www.ncbi.nlm.nih.gov/sra/PRJNA702123) and PRJNA751406 (https://www.ncbi.nlm.nih.gov/sra/PRJNA751406), respectively.

## Abbreviations

ABA: Abscisic acid
APX: Ascorbate peroxidase
ATP: Adenosine-triphosphate
CAT: Catalase
DBG: De Bruijn Graph
DEG: Differentially expressed gene
eggNOG: Evolutionary genealogy of genes: non-supervised orthologous groups
FPKM: Fragments per kilobase of exon model per million mapped reads
GO: Gene ontology
H_2_O_2_: Hydrogen peroxide
HSF: Heat stress transcription factor
HSP: Heat shock protein
JA: Jasmonic acid
KEGG: Kyoto encyclopedia of genes and genomes
NADPH: Nicotinamide adenine dinucleotide phosphate
NGS: Next generation sequencing
NR: NCBI non-redundant
Pn: Net photosynthetic rate
POD: Peroxidase
PSI: Photosystem I
PSII: Photosystem II
ROS: Reactive oxygen species
SA: Salicylic acid
SOD: Superoxide dismutase
TMV: Anti-tobacco mosaic virus
TR: Transpiration rate
