# Correction to: Comparative transcriptome analysis of heat stress responses of *Clematis lanuginosa* and *Clematis crassifolia*

**DOI:** 10.1186/s12870-022-03566-0

**Published:** 2022-04-05

**Authors:** Renjuan Qian, Qingdi Hu, Xiaohua Ma, Xule Zhang, Youju Ye, Hongjian Liu, Handong Gao, Jian Zheng

**Affiliations:** 1 College of Forestry, Nanjing Forestry University, Nanjing, 210037 China; 2 Wenzhou Key Laboratory of Resource Plant Innovation and Utilization, Zhejiang Institute of Subtropical Crops, Wenzhou, 325005 Zhejiang China; 3 Zhejiang Institute of Subtropical Crops, Zhejiang Academy of Agricultural Sciences, Wenzhou, 310021 Zhejiang China

**Correction to: BMC Plant Biol 22, 138 (2022)**

**https://doi.org/10.1186/s12870-022-03497-w**

Following publication of the original article [1], authors would like to correct Affiliation 1 details. Below is the correct affiliation:

^1 ^College of Forestry, Nanjing Forestry University, Nanjing 210037, China.

The original article has been corrected.

## References

[1] Qian R, Hu Q, Ma X (2022). Comparative transcriptome analysis of heat stress responses of *Clematis lanuginosa* and *Clematis crassifolia*. BMC Plant Biol.

