# Supplementary material for: Comparative transcriptome analysis of heat stress responses of Clematis lanuginosa and Clematis crassifolia
Source: BMC Plant Biol. 2022 Mar 23;22:138. doi: 10.1186/s12870-022-03497-w (PMC8941805; doi:10.1186/s12870-022-03497-w)
Supplement: Supplementary file 1 — Additional file 1: Fig. S1. Gene Ontology classification and KEGG analysis of the transcriptome. Fig. S2. Enriched GO terms of DEGs. Fig. S3. Enriched GO terms (cellular component) (P < 0.05) of DEGs. Fig. S4. Clustering analysis of gene expression. [file 12870_2022_3497_MOESM1_ESM.docx]

**
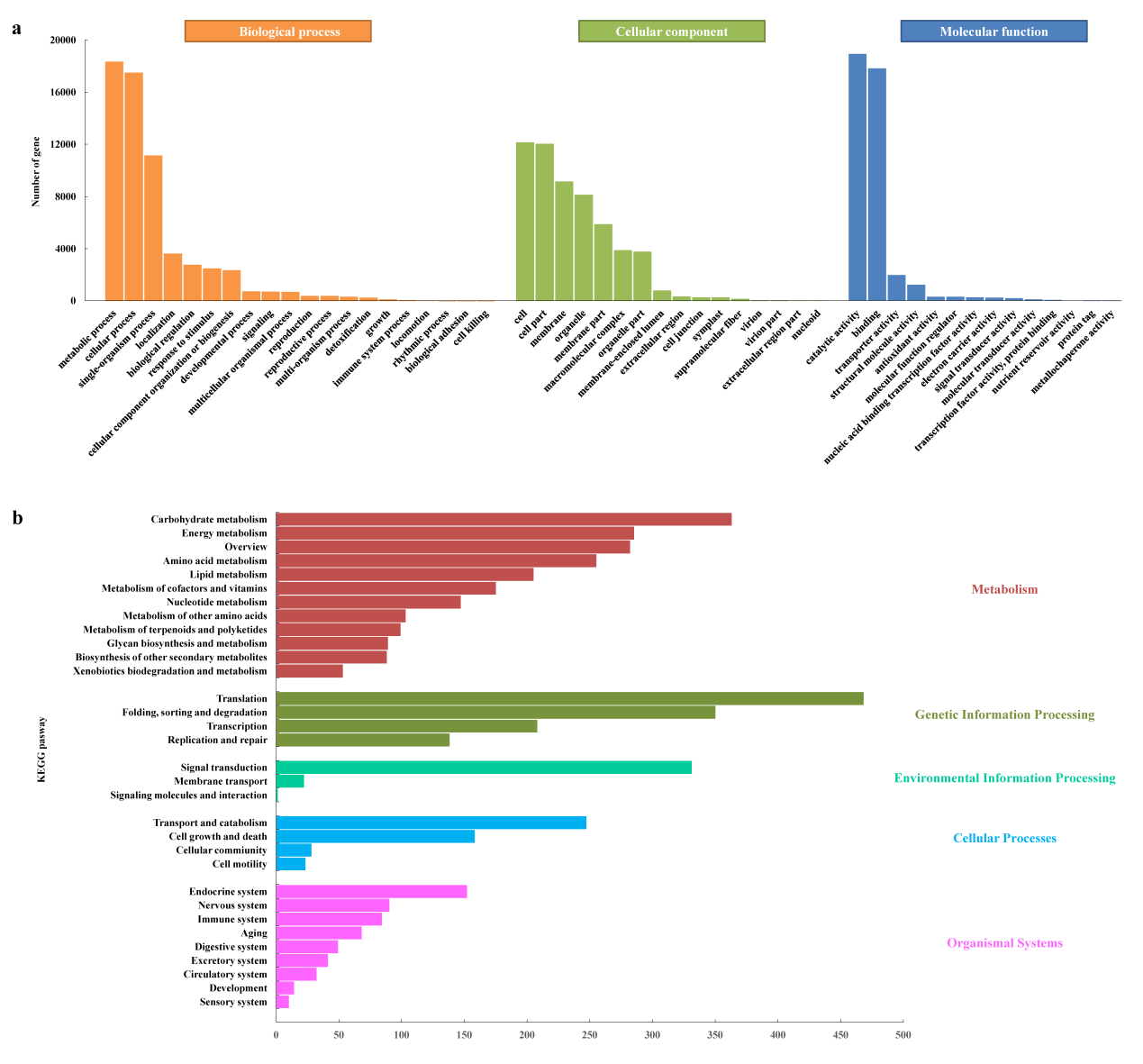
**

**Fig. S1 Gene Ontology classification and KEGG analysis of the transcriptome.** **a** Annotation of the GO function of the transcript. **b** Annotation of the KEGG function of the transcript.





**Fig. S2 Enriched GO terms of DEGs.**





**Fig. S3 Enriched GO terms (cellular component) (P < 0.05) of DEGs. a** *C. lanuginosa;* **b** *C. crassifolia*.


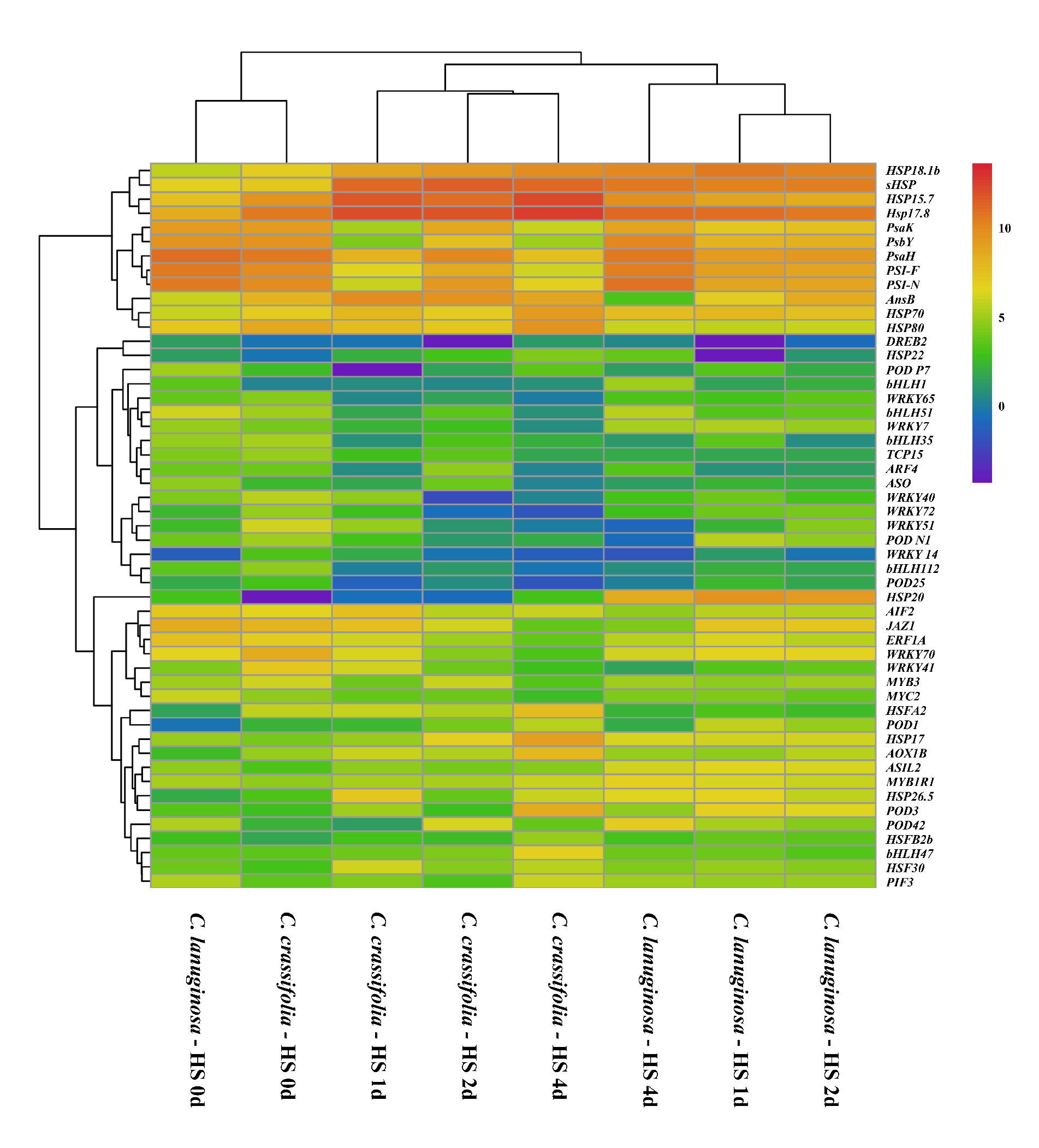


**Fig. S4** Clustering analysis of gene expression.
