# Supplementary material for: Comparative transcriptome analysis of heat stress responses of Clematis lanuginosa and Clematis crassifolia
Source: BMC Plant Biol. 2022 Mar 23;22:138. doi: 10.1186/s12870-022-03497-w (PMC8941805; doi:10.1186/s12870-022-03497-w)
Supplement: Supplementary file 2 — Additional file 2: Table S1. Statistics of splicing results. Table S2. Annotation of unique DEGs related to heat stress in C. lanuginose. Table S3. Annotation of unique DEGs related to heat stress in C. crassifolia. Table S4. The GenBank number of the unigenes. Table S5. Primer sequences for gene expression analysis. [file 12870_2022_3497_MOESM2_ESM.docx]

**Table S1**. Statistics of splicing results.

| Statistics | Contig | Transcript | Unigene |
| --- | --- | --- | --- |
| Total Length (bp) | 272054542 | 278886193 | 167820823 |
| Sequence Number | 1086589 | 540495 | 395844 |
| Max. Length (bp) | 16844 | 14000 | 14000 |
| Mean Length (bp) | 250.3748354 | 515.9829286 | 423.9569704 |
| N50 (bp) | 254 | 703 | 447 |
| N50 Sequence No. | 246259 | 100682 | 83065 |

**Table S2.** Annotation of unique DEGs related to heat stress in *C. lanuginose.*

| Gene Code | GenBank No. | Description |
| --- | --- | --- |
| c198500_g2 | gi\|635658982 | CBF/DREB1 transcription factor 1 |
| c210721_g1 | gi\|222431915 | Heat shock protein 90A |
| c198998_g1 | gi\|552546120 | Cytochrome b6 |
| c186820_g1 | gi\|343222 | Cytochrome oxidase subunit I |
| c175146_g2 | gi\|926787024 | Alcohol dehydrogenase groes domain protein |
| c152743_g1 | gi\|590613857 | Aldehyde dehydrogenase 5F1 |
| c177731_g1 | gi\|255088325 | Aldehyde reductase |
| c196685_g1 | gi\|658309386 | Anthocyanidin reductase |
| c208623_g2 | gi\|595597722 | CAC1 protein |
| c172261_g1 | gi\|971506369 | Carboxymethylenebutenolidase |
| c119533_g1, c183734_g1 | gi\|971505636 | Catalase |
| c211776_g3 | gi\|325551315 | Chalcone isomerase |
| c188872_g1 | gi\|302844044 | Component of cytosolic 80S ribosome and 60S large subunit |
| c209933_g1 | gi\|590601187 | Gigantea protein isoform 1 |
| c211018_g1 | gi\|675272222 | Glycosyltransferase |
| c170656_g1 | gi\|573025883 | Iridoid synthase |
| c203365_g3 | gi\|403406446 | Leucoanthocyanidin reductase |
| c190253_g1 | gi\|922336447 | Lipid transfer protein |
| c177280_g1 | gi\|6469139 | Malate dehydrogenase |
| c206863_g1 | gi\|590723158 | Pectinesterase 2 |
| c184624_g1 | gi\|930809512 | Polyphenol oxidase |
| c186866_g1 | gi\|971512632 | Potassium channel beta subunit 1 |
| c204326_g1 | gi\|72256517 | Pyridoxine biosynthesis protein |
| c194617_g1 | gi\|29725558 | SNF1 kinase complex anchoring protein |
| c198590_g1 | gi\|526117816 | Sucrose responsive element binding protein |
| c187021_g1 | gi\|28193048 | SUI1 protein |
| c208155_g2 | gi\|313474921 | Tetrahydroberberine oxidase |
| c198695_g1 | gi\|926781046 | Thiamine biosynthetic enzyme |
| c210055_g1 | gi\|590625828 | Thiazole biosynthetic enzyme |
| c207138_g2, c179416_g1 | gi\|246215 | Thionin |
| c203169_g1 | gi\|590696668 | Thioredoxin superfamily protein isoform 1 |
| c210101_g1 | gi\|469474163 | Transketolase |

**Table S3.** Annotation of unique DEGs related to heat stress in *C. crassifolia.*

| Gene Code | GenBank No. | Description |
| --- | --- | --- |
| c202884_g2 | gi\|703100592 | ABC transporter |
| c201860_g1 | gi\|703098266 | Nitrate transporter 1. |
| c183648_g1 | gi\|312201055 | Potassium uptake transporter 2 |
| c195956_g1 | gi\|526117878 | Transcription factor APETALA2 |
| c189120_g1 | gi\|836634238 | WRKY transcription factor |
| c192857_g1, c194731_g1 | gi\|524908120 | WRKY3 transcription factor |
| c202733_g1 | gi\|590648611 | Zinc finger protein 4 |
| c210368_g1 | gi\|948299595 | Photosystem II 44 kda protein |
| c208712_g3 | gi\|944542068 | Photosystem II protein D |
| c192904_g1 | gi\|1032297090 | Psab |
| c195700_g1 | gi\|590705619 | Acetamidase/Formamidase family protein isoform 1 |
| c205322_g9 | gi\|27527741 | Aldehyde dehydrogenase |
| c182789_g1 | gi\|590666546 | Annexin 5 isoform 1 |
| c198184_g2 | gi\|429465362 | ATP synthase subunit 1 |
| c203736_g1 | gi\|58530710 | Ethylene receptor |
| c204211_g3 | gi\|675272220 | Glycosyltransferase |
| c184173_g1 | gi\|391349165 | Histidine phosphotransfer protein |
| c186710_g1 | gi\|118490068 | Lipid transfer protein isoform 1.1 precursor |
| c205447_g1 | gi\|590578151 | Major facilitator superfamily protein isoform 2 |
| c104038_g1 | gi\|590669586 | Pectate lyase family protein isoform 3 |
| c205659_g7 | gi\|336239287 | Photosystem II CP47 chlorophyll apoprotein |
| c210114_g2 | gi\|590687719 | Polyol/monosaccharide transporter 5 |
| c187057_g1 | gi\|696196923 | Proteinase inhibitor 1 |
| c208783_g1 | gi\|566190401 | Sulfate transporter 3.3 family protein |
| c196580_g1 | gi\|741207319 | UDP glucose: glycoprotein glucosyltransferase protein |
| c112993_g1 | gi\|590588699 | UGT protein isoform 2 |
| c211423_g1 | gi\|703121465 | Xanthine dehydrogenase |
| c192732_g1 | gi\|340396652 | Xyloglucan endotransglucosylase/hydrolase |

**Table S4.** The GenBank number of the unigenes.

| Gene Code | GenBank No. | Description |  | Gene Code | GenBank No. | Description |
| --- | --- | --- | --- | --- | --- | --- |
| c208692_g2 | AIF2 | gi\|702473348 |  | c203219_g1 | WRKY72 | gi\|719965476 |
| c197707_g1 | ARF4 | gi\|720027964 |  | c188534_g1 | HSP15.7 | gi\|720049054 |
| c195867_g1 | ASIL2 | gi\|720059207 |  | c192936_g1 | HSP17 | gi\|225462326 |
| c205626_g1 | bHLH1 | gi\|720000103 |  | c176964_g1 | HSP17.8 | gi\|1009121224 |
| c197850_g1 | bHLH35 | gi\|357449601 |  | c199407_g2 | HSP18.1b | gi\|315932728 |
| c200349_g1 | bHLH47 | gi\|720007721 |  | c201522_g2 | HSP20 | gi\|590603634 |
| c196189_g1 | bHLH51 | gi\|719993189 |  | c190633_g4 | HSP22 | gi\|1024052703 |
| c203571_g1 | bHLH112 | gi\|720038065 |  | c200771_g1 | HSP26.5 | gi\|1028972941 |
| c182557_g1 | DREB2 | gi\|819320934 |  | c204924_g1 | HSP70 | gi\|702444682 |
| c192111_g1 | ERF1A | gi\|826136607 |  | c196872_g2 | HSP80 | gi\|1031985647 |
| c206233_g2 | HSFA2 | gi\|147866670 |  | c192391_g1 | sHSP | gi\|590674502 |
| c194434_g1 | HSFB2b | gi\|720073097 |  | c200656_g1 | AnsB | gi\|823165645 |
| c194517_g1 | HSF30 | gi\|720019372 |  | c206127_g1 | AOX1B | gi\|590680704 |
| c194555_g1 | JAZ1 | gi\|348076045 |  | c209528_g1 | ASO | gi\|694437085 |
| c204139_g1 | MYB1R1 | gi\|719965160 |  | c200317_g1 | POD 1 | gi\|28629828 |
| c198261_g1 | MYB3 | gi\|313474116 |  | c210145_g2 | POD 3 | gi\|720077128 |
| c208293_g1 | MYC2 | gi\|225427201 |  | c188600_g1 | POD 25 | gi\|802640387 |
| c209598_g2 | PIF3 | gi\|720048280 |  | c199977_g2 | POD 42 | gi\|816206858 |
| c195225_g2 | TCP15 | gi\|719965639 |  | c203256_g1 | POD N1 | gi\|590688650 |
| c206794_g2 | WRKY7 | gi\|225438803 |  | c204229_g1 | POD P7 | gi\|1026036834 |
| c198702_g1 | WRKY14 | gi\|225444177 |  | c188817_g1 | PsaH | gi\|719980139 |
| c189120_g1 | WRKY40 | gi\|836634238 |  | c191077_g1 | PsaK | gi\|720088668 |
| c200654_g1 | WRKY41 | gi\|720033546 |  | c200811_g3 | PsbY | gi\|224110818 |
| c187717_g1 | WRKY51 | gi\|720016168 |  | c195016_g3 | PSI-F | gi\|1026026065 |
| c195073_g1 | WRKY65 | gi\|720080894 |  | c187820_g1 | PSI-N | gi\|720046035 |
| c195444_g1 | WRKY70 | gi\|719990708 |  |  |  |  |

**Table S5**. Primer sequences for gene expression analysis.

| Gene code | Gene description | Primer |
| --- | --- | --- |
| *GAPDH*-F | *GAPDH* | AACCCTGAGGAGATTCCA |
| *GAPDH*-R |  | CACCACCCTTCAAGTGAGCAG |
| *c192936_g1*-F | *HSP17* | GGAGAAAGCACTGAAGAAGAAG |
| *c192936_g1*-R |  | ACAACACACAAAACGACCAA |
| *c176964_g1*-F | *HSP17.8* | AACAACGACCCCTTCTCC |
| *c176964_g1*-R |  | CCTCCACCTTCACTTCCTC |
| *c200771_g1*-F | *HSP26.5* | CATCAAGGGAGAGCACAAG |
| *c200771_g1*-R |  | AACACCATCTTTCATTTCGG |
| *c204924_g1*-F | *HSP70* | AGTTTCATTCAGTCGCTTCC |
| *c204924_g1*-R |  | CAATCTTCTCGCTTCCACTAC |
| *c199407_g2*-F | *HSP18.1* | TCTCCATTCCTCTACCAACTTCAT |
| *c199407_g2*-R |  | TTCCACCTTCACCTCTTCCTT |
| *c201522_g2*-F | *HSP20* | GCATAGAGTGGAGCGTTCTTAC |
| *c201522_g2*-R |  | TGATCTGGTCTGGTGACAACT |
| *c206233_g2*-F | *HSFA2* | AGGAGGGTGATGTTGTGG |
| *c206233_g2*-R |  | TGGGAAGAAGAGTGGTTGA |
| *c182557_g1*-F | *DREB2* | GTGCATGAAAGGAAAAGGAG |
| *c182557_g1*-R |  | CAACCCATTTACCCCAAGT |
| *c194555_g1*-F | *JAZ1* | GGAGGAGAAGGTGAAGGAG |
| *c194555_g1*-R |  | GGAGGAGAAGGTGAAGGAG |
| *c200317_g1*-F | *POD1* | CTCATACATTGGGCTTTTCTC |
| *c200317_g1*-R |  | CAACTACCTTCGGGCATT |
| *c210145_g2*-F | *POD3* | TCCTCCACCAACAGAAAACT |
| *c210145_g2*-R |  | GGTCTACATCTCCATTCCCA |
